# Supplementary material for: Phospho-DIGE Identified Phosphoproteins Involved in Pathways Related to Tumour Growth in Endometrial Cancer
Source: Int J Mol Sci. 2023 Jul 26;24(15):11987. doi: 10.3390/ijms241511987 (PMC10419128; doi:10.3390/ijms241511987)

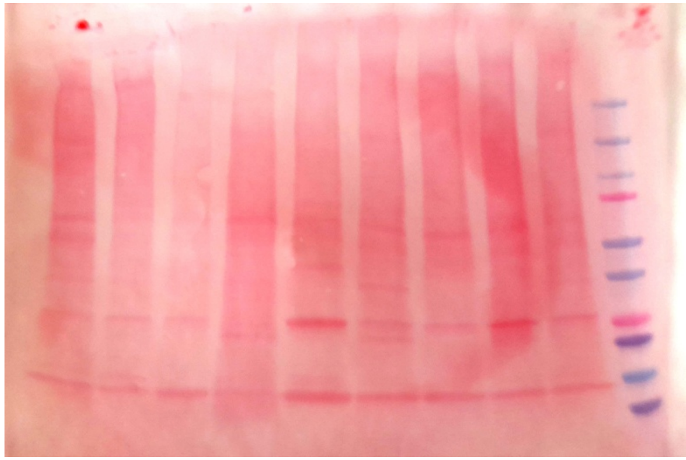

Gel 2

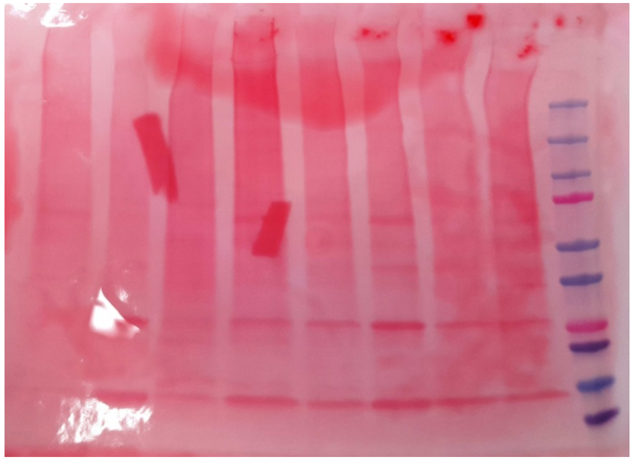

Gel 1

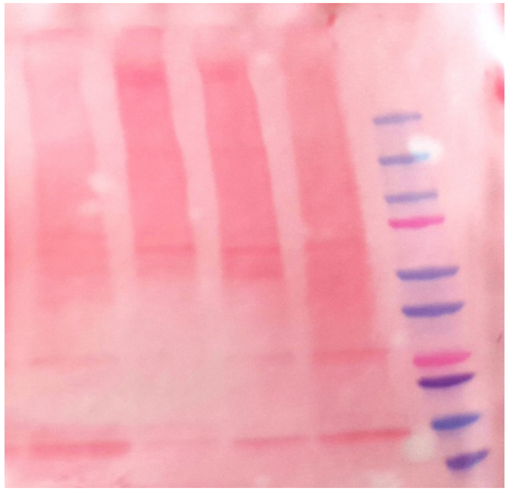

Gel 4

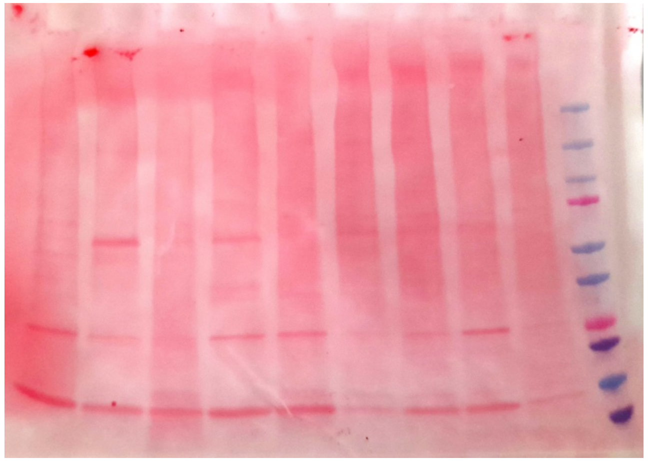

Gel 3

Whole membrane stained with red ponceau

Whole membrane of HBB protein

C-control
EC- endometrial cancer

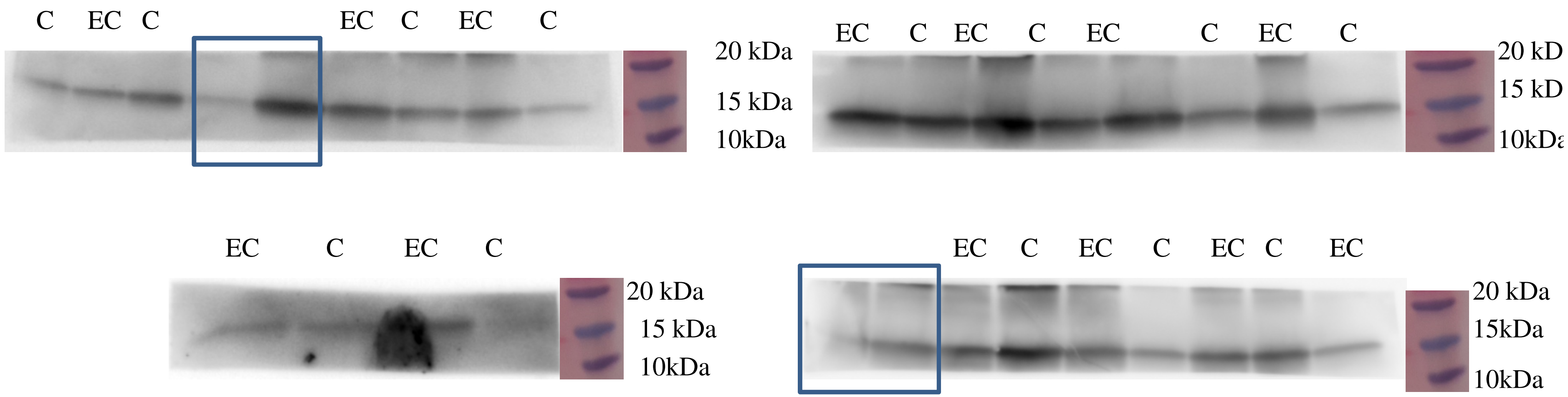

Whole membrane of HSPB1 protein

C-control
EC- endometrial cancer

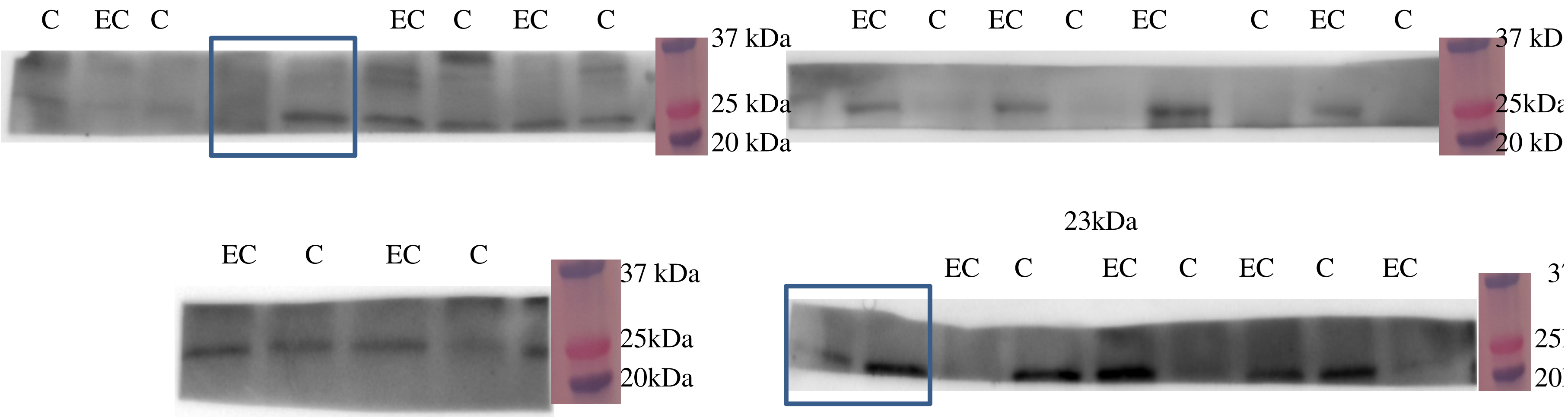

Whole membrane of CKB protein

C-control
EC- endometrial cancer

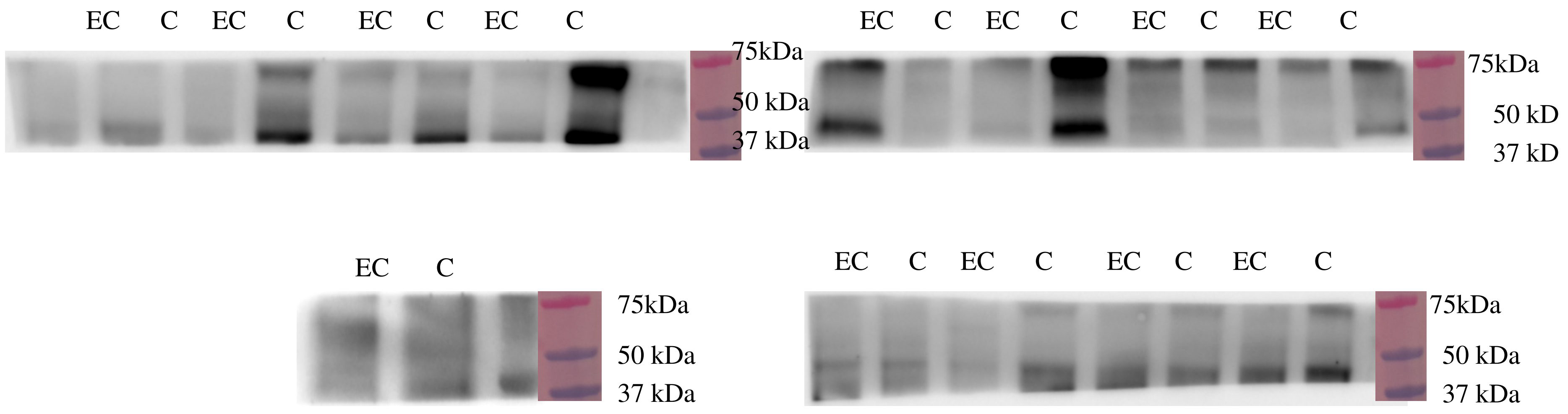

Whole membrane of LDHB protein

C-control
EC- endometrial cancer

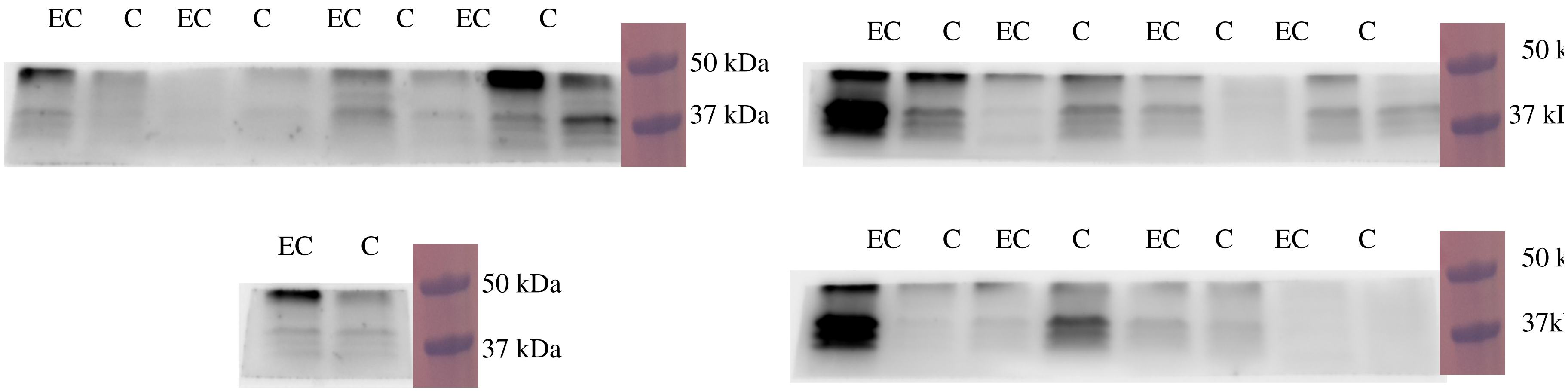

Supplement: Supplementary file 1 [file ijms-24-11987-s001.zip › Figure S1.pdf]
