# Supplementary material for: Phospho-DIGE Identified Phosphoproteins Involved in Pathways Related to Tumour Growth in Endometrial Cancer
Source: Int J Mol Sci. 2023 Jul 26;24(15):11987. doi: 10.3390/ijms241511987 (PMC10419128; doi:10.3390/ijms241511987)
Supplement: Supplementary file 1 [file ijms-24-11987-s001.zip › Supplementary Table S1.pdf]

Sample type	Age	Diagnosis	Grade	Stage	Biological sources	Methodological used
Tumor	74	Endometroid adenocarcinoma	G2	IB	Tissue	2D-DIGE and western-blotting
Tumor	59	Endometroid adenocarcinoma	G1	IA	Tissue	2D-DIGE and western blotting
Tumor	79	Endometroid adenocarcinoma	G1	IB	Tissue	2D-DIGE and western blotting
Tumor	76	Endometroid adenocarcinoma	G2	IB	Tissue	2D-DIGE and western blotting
Tumor	57	Endometroid adenocarcinoma	G2	IB	Tissue	2D-DIGE and western blotting
Tumor	78	Endometroid adenocarcinoma	G2	IB	Tissue	2D-DIGE and western blotting
Tumor	73	Endometroid adenocarcinoma	G2	IA	Tissue	2D-DIGE and western blotting
Tumor	70	Endometroid adenocarcinoma	G1	IB	Tissue	2D-DIGE and western blotting
Tumor	53	Endometroid adenocarcinoma	G1	IA	Tissue	Wester blotting
Tumor	67	Endometroid adenocarcinoma	G1	IB	Tissue	Western blotting
Tumor	55	Endometroid adenocarcinoma	G2	IA	Tissue	Western blotting LC-MS/MS
Tumor	71	Endometroid adenocarcinoma	G3	IA	Tissue	Western blotting LC/MS-MS
Tumor	68	Endometroid adenocarcinoma	G1	IB	Tissue	Western blotting LC/MS-MS
Control	47	Leiomyoma			Tissue	2D-DIGE and western-blotting
Control	42	Leiomyoma			Tissue	2D-DIGE and western blotting
Control	45	Leiomyoma			Tissue	2D-DIGE and western blotting
Control	40	Leiomyoma			Tissue	2D-DIGE and western blotting

Control	32	Leiomyoma			Tissue	2D-DIGE and western blotting
Control	44	Leiomyoma			Tissue	2D-DIGE and western blotting
Control	47	Leiomyoma			Tissue	2D-DIGE and western blotting
Control	42	Leiomyoma			Tissue	2D-DIGE and western blotting
Control	47	Leiomyoma			Tissue	Wester blotting
Control	41	Leiomyoma			Tissue	Western blotting
Control	51	Leiomyoma			Tissue	Western blotting
Control	42	Leiomyoma			Tissue	Western blotting
Control	45	Leiomyoma			Tissue	Western blotting

Supplementary Table S1: Clinico-pathological characteristics of the 26 women enrolled in the study
